# Parkinsonism: A Rare Adverse Effect of Valproic Acid

**DOI:** 10.7759/cureus.8782

**Published:** 2020-06-23

**Authors:** Abilash Muralidharan, Jawaria Rahman, Dipanjan Banerjee, Abdul Rub Hakim Mohammed, Bilal Haider Malik

**Affiliations:** 1 Internal Medicine, California Institute of Behavioral Neurosciences and Psychology, Fairfield, USA; 2 Internal Medicine, Kiruba Hospital, Coimbatore, IND; 3 Pathology, City of Hope Comprehensive Cancer Center, Monrovia, USA; 4 Neuroscience, California Institute of Behavioral Neurosciences and Psychology, Fairfield, USA; 5 Geriatrics, Queen's Medical Center, Nottingham University Hospitals NHS Trust, Nottingham, GBR

**Keywords:** valproic acid, parkinsonism, vaproic acid induced parkinsonism, drug induced parkinsonism

## Abstract

Valproic acid (VPA) is an anti-epileptic drug (AED) used as a first-choice agent for most forms of epilepsy. It is used in the treatment of manic episodes, bipolar disorder, migraine prevention, and impulse control. Hence it is one of the most commonly prescribed drugs by physicians nowadays. VPA acts by increasing gama amino butyric acid (GABA) levels, and also reduces neuronal activation by blocking voltage-gated sodium, potassium, and calcium channels. VPA has various adverse effects like thrombocytopenia, hyperammonemia, teratogenicity causing spina bifida in newborns when exposed in utero. The focus of this review is to research one such easily overlooked adverse effect of VPA, which is VPA-induced Parkinsonism.

We carried out a review of literature and gathered all comprehensive peer-reviewed articles from PubMed. The data for this research were collected ethically and legally after a thorough examination of the literature.

Data obtained from the studies have suggested that Parkinsonism is an adverse effect of VPA. Chronic usage of VPA causes Parkinsonism. It occurs equally in males and females, more common in older people usually above the age of 55 years and not dose-dependent. According to the data obtained, all patients who developed Parkinsonism had serum levels in the therapeutic range (50-100 mcg/mL). Thus the chronic intake of maintenance dose of VPA seems to be the leading cause. The symptoms usually improve over a few weeks and fully resolve in a few months after stopping the drug. When the patient's symptoms do not improve, it means VPA has unmasked the underlying potential for developing Parkinson's disease. Such patients benefit from levodopa therapy. However, the mechanism of how VPA causes Parkinsonism remains unknown.

Based on the articles reviewed, we hypothesize that VPA’s mechanism of neuronal inactivation by blocking membrane channels across the neuronal membrane, primarily when used chronically could be the mechanism by which it causes Parkinsonism. VPA causes down regulation of sodium and potassium channels on neuronal membrane in order to stop the neurons from firing. Thereby a decrease in action potential across the neurons causes a temporary physiological inactivation of the neuron. When multiple neurons are inactivated in the basal ganglia of the brain, the patient develops symptoms of Parkinsonism. As the neurons are only temporarily inactivated physiologically, when the drug is stopped the membrane receptors are reactivated on the neuronal membranes. This leads to neuronal activation and neuronal membrane potential becomes the same as before. The above mechanism clarifies why the symptoms settle down when the medication is stopped.

## Introduction and background

“Parkinson’s is a slow but inevitable process. It’s hard living with it on a daily basis. The difficulty facing people with it is that they never quite know ‘Can I or Can’t I do this today”

- Helen Mirren [1].

According to the statistics by the Parkinson’s Foundation, approximately 60,000 people are newly diagnosed with Parkinson's disease every year. There are 10 million people worldwide living with Parkinson’s disease. The cost of Parkinson's, including the treatment, social security payments, and lost income is estimated to be about $52 billion per year in the United States alone [2]. The above statistics clearly show how much of a burden Parkinson’s disease is to the person, his family, and the nation.

Parkinson’s disease is a degenerative disorder of the nervous system due to the loss of the dopaminergic neurotransmitter system within the substantia nigra (SN) of the brain [3]. It occurs due to dominant mutations in the α‐synuclein gene, a major protein component of Lewy bodies [4-5]. Parkinson’s disease is characterized by bradykinesia, resting tremor, rigidity, and postural instability. The patients also have a wide range of cognitive impairments like fluctuating consciousness, executive function deficits, and memory deficits [6-7]. They also suffer from a wide range of nonmotor symptoms that precede the characteristic movement disorder such as hyposmia, sleep disturbances (REM sleep disorder), and depression [8]. Parkinson’s disease also causes visual dysfunction by affecting visual acuity, dynamic contrast sensitivity, color discrimination, visuospatial orientation dysfunction, facial recognition problems, abnormal stereopsis, and chronic visual hallucinations [9-10]. The diagnosis of Parkinson's disease is based on clinical features. It is also based on the response to dopamine agents and the development of motor fluctuations [11]. Diagnostic criteria used for diagnosing Parkinson’s disease is The Movement Disorder Society (MDS) criteria for diagnosing Parkinson’s disease, which has a very high specificity but limited sensitivity. It is used more often for research than for clinical diagnosis of Parkinson’s disease [12]. Another criteria commonly used for clinically diagnosing Parkinson’s disease with a diagnostic accuracy of up to 90% is the UK Parkinson’s disease society brain bank criteria [13]. Life expectancy is decreased in Parkinson’s disease. The odds ratio is 2.56 (the mortality risk is 2.56 times higher than similar age-matched people without Parkinson’s disease) [14]. Many reports have shown that Parkinsonism can develop when exposed to environmental toxins like 1‐methyl‐4‐phenyl‐1,2,3,6‐tetrahydropyridine (MPTP - a by-product in the manufacture of meperidine), insecticides like paraquat and rotenone, and solvents such as trichloroethylene and perchloroethylene [15]. The various drugs that cause Parkinsonism include anti-psychotics (most common), antidepressants, calcium channel antagonists, gastrointestinal prokinetics, and anti-epileptic drugs (AEDs) [16].

The term seizure is derived from the Latin word sacire, meaning "to take possession of." It is a sudden event that occurs due to abnormal neuronal activity in the brain [17]. Depending on the neurons involved, seizure can have various manifestations. Epilepsy occurs when the patient has recurrent seizures due to genetic inheritance and structural abnormalities of the brain [18-19]. The estimated average incidence of epilepsy each year in the United States is 48 for every 100,000 people. One in 26 people all over the world develop epilepsy at some point in their life [20]. Valproic acid (VPA) is a broad-spectrum AED that is generally regarded as a first-choice agent for most forms of idiopathic and symptomatic generalized epilepsies [21]. VPA, which is chemically 2-propylpentanoic acid is a branched short-chain fatty acid (SCFA) made from valeric acid. It is highly protein-bound (87%-95%), resulting in low clearance (6-20 mL/h/kg) [22].

The VPA metabolism in humans occurs in three ways: glucuronidation, β oxidation in the mitochondria, and cytochrome P450 (CYP)-mediated oxidation [23]. Impairment of GABA inhibitory activity can lead to convulsions. VPA action on neurons to prevent epilepsy is by increasing gamma amino butyric acid (GABA) levels and by reducing the high-frequency firing of neurons by blocking voltage-gated sodium, potassium, and calcium channels [24]. VPA was demonstrated to be an inhibitor of histone deacetylase 1 (HDAC) as well as other HDACs. It potentially increases the expression of genes involved in apoptosis and anti-tumor action, so it has been proposed to be a potential anti-tumor agent [25]. VPA has various adverse effects -- a significant decrease in platelet count and an increased risk for developing hyperammonemia. It also causes an increase in triglyceride levels and risk of developing pituitary-thyroid axis dysregulation. VPA causes teratogenicity by causing spina bifida in newborns, especially when there is an intake of the drug during the first trimester of pregnancy. It is also reported to cause behavioral disorders like autism in children when there is in utero exposure to VPA in second and third trimesters [26-29]. These are the most common adverse effects of long-term VPA treatment, especially in adolescent and young adult psychiatric patients.

In this review, we focus on a potential adverse effect of VPA, which is Parkinsonism. Very few cases have been reported. There is less literature available regarding VPA-induced Parkinsonism.

## Review

Methods and results

A search was comprehensively carried out on PubMed to conduct a detailed study on whether VPA causes Parkinsonism. All the data were collected after a thorough review of the literature. Preferred Reporting Items for Systematic Reviews and Meta-Analysis (PRISMA) guidelines, quality assessment tools, and statistical analysis were not used as this was a traditional review article. The whole data were collected legally and ethically. Studies explicitly done on humans were included in this study. Only peer-reviewed articles were included in this study.

This traditional review article search was accomplished by using three keywords. Table 1 contains the keyword used for search, the database used for search, and the number of literatures obtained. A search with VPA as a keyword showed 5856 results; a search with Parkinsonism as a keyword showed 59668 results; a search with VPA induced Parkinsonism yielded only 71 results, clearly showing the lack of research in that topic.

**Table 1:** Keywords used, database used, and number of literatures available. VPA, Valproic acid

Keyword	Database	Number of literatures
VPA	PubMed	5856
Parkinsonism	PubMed	59668
VPA-induced Parkinsonism	PubMed	71

Discussion

Valproic Acid

Valproic acid is a drug approved by the Food and Drug Administration (FDA) for the use as a treatment modality in various seizure disorders and epilepsy prophylaxis. In recent years it has been approved for treating manic episodes, bipolar disorder, and also for migraine prophylaxis. VPA is also used off the label to treat psychiatric conditions like impulse control and post traumatic stress disorders (PTSD) [30-32]. Recent researches have shown the effect of VPA on histone deacetylase (HDAC) molecules. So it is being considered as an anti-tumor chemotherapeutic agent for the treatment of breast cancer [33]. Because it has a broad spectrum of seizure coverage, it is the most commonly prescribed anti-epileptic drug. There is a common saying amongst neurologists that if a patient has a seizure, you can blindly prescribe him VPA without even having to diagnose the type of seizure, and the patient would improve. Because of the uses as mentioned above, VPA is one of the most commonly prescribed drugs by physicians, especially neurologists and psychiatrists.

Drug-Induced Parkinsonism 

After idiopathic Parkinson's disease (IPD), drug-induced Parkinsonism (DIP) is more common in developed countries like the United States and Japan [34]. The incidence rate of DIP is 3.3 per 100,000 person-years, higher in women, and increases with old age [35]. The incidence of DIP has risen since 2012 [36]. Risk factors for DIP include old age ( > 55 years), dose and duration of treatment, type of drug used, cognitive impairment, AIDS, tardive dyskinesia, and pre-existing extrapyramidal disorder [37-39]. Previously during the 1980s, drugs like cinnarizine and flunarizine were the drugs commonly causing DIP. In recent years the usage of these drugs has become rare. Hence they have been replaced by drugs like gastrointestinal prokinetic agents like metoclopramide, anti-psychotics, antiemetics, antidepressants, calcium channel blockers, and anti-epileptics. Exposure to environmental toxins like 1‐methyl‐4‐phenyl‐1,2,3,6‐tetrahydropyridine (MPTP- a by-product in the manufacture of meperidine), insecticides like paraquat and rotenone, solvents such as trichloroethylene and perchloroethylene also causes symptoms of Parkinsonism in people [3]. The symptoms and clinical features of DIP is similar to Parkinson's disease. They include bradykinesia, resting tremor, rigidity, postural instability, and a wide range of cognitive impairment. The main difference between Parkinson’s disease and DIP is the presence of hyposmia. It is specific for DIP [40]. Parkinson's disease is a prolonged disease process and usually takes many years before the symptoms become apparent. It takes many more years till the patient has the full-blown disease. DIP is a rapidly progressive disease. Patients with DIP develop severity in a matter of a few days after the initial symptoms occur [41]. The diagnosis of DIP is based on careful history obtained from the patient about the drugs he has been taking ruling out any environmental exposures that could have caused the symptoms and, finally, neurological examination [42-43]. Diagnostic imaging studies like dopamine transporter imaging (DTI) can be used to differentiate Parkinson’s disease and DPI [44]. Preliminary evidence exists for sympathetic cardiac scintigraphy (SCS) which is an investigative tool that can be used to predict dopaminergic pathway abnormalities. It helps to distinguish between DIP and Parkinson's disease. Out of the above-mentioned diagnostic studies, only imaging of the dopamine pathway remains the only feasible and reasonably available technique for differentiating Parkinson’s disease and DIP. Diagnostic criteria like MDS criteria for Parkinson’s disease and UK Parkinson’s disease society brain bank for Parkinsonism can be used to diagnose DIP based on symptoms alone [45]. The treatment for DIP is stopping the drug causing it. If the medication cannot be withdrawn, then he should be switched to a drug class with a lesser risk of DIP. There is no evidence suggesting that usage of levodopa and anticholinergic has benefit when prescribed to patients with DIP. Parkinsonism symptoms usually improve over several weeks. The symptoms typically stop entirely within several months after stopping the drug. In some patients, the symptoms may occur for a long time despite stopping the offending medication. In such situations, we need to consider the drug was unmasking an underlying potential to develop Parkinson’s disease. In such circumstances, the usage of anti-Parkinson’s medications may help the patient. The main problem with DIP is that despite being a significant health problem in specific populations, it seems to be frequently overlooked by the medical community. Prescribers should be vigilant in the elderly and patients taking multiple drugs for DIP.

Valproic Acid Induced Parkinsonism 

The most common adverse effects of VPA include nausea, diarrhea, and vomiting. The most common neurological adverse effects include sedation, ataxia, and tremor. Many cases have been reported recently regarding VPA's ability to cause a reversible form of Parkinsonism [46].

Silver et al. reported five cases in which the patient who was on long-term VPA therapy developed symptoms of Parkinsonism. Parkinsonism was defined by the development of bradykinesia, rigidity, postural instability, and resting tremor. The patient’s symptoms improved upon the stoppage of the drug. It took a few months for the symptoms to resolve completely. In one patient, there was the unmasking of underlying Parkinson's disease that occurred. That patient benefitted from levodopa therapy [47].

Mahmoud and Tampi described a case in which the patient developed a severely disabling akinetic rigid syndrome when he was put on chronic valproate therapy. The symptoms improved partially when the drug was stopped. The patient was then put on dopaminergic medications, which showed good improvement of symptoms. They hypothesized that unmasking of underlying clinically silent Parkinson's disease was done by high doses of VPA [48].

Masmoudi et al. reported 10 cases in his research with a proportionate incidence of VPA-induced Parkinsonism in males and females. All patients were on chronic use of VPA over many years before developing the symptoms. The serum levels of VPA were measured in all the patients when they developed Parkinsonism. The drug level was within the therapeutic range (50-100 mcg/L) in all the patients. This clearly shows that the development of Parkinsonism does not depend on the dose or dosage of the drug. It depends on the chronic usage of the drug [49].

Park and Tazawa reported three cases in which there was a development of Parkinsonism in elderly patients due to chronic usage of VPA. The first case was a 75-year-old woman who developed symptoms of Parkinsonism after VPA usage for 41 months at 800 mg/day dosage. The second case was a 70-year-old man who developed symptoms of Parkinsonism after VPA usage for 15 months at 800 mg/day. The third case was that of a 74-year-old woman who developed Parkinsonism symptoms after 14 months of ingesting VPA at 800 mg/day. They hypothesized that old age, prolonged duration of treatment, and maintenance of a therapeutic range of the drug throughout the extended period of therapy could be risk factors for VPA-induced Parkinsonism. The incidence of DIP occurs in young individuals (<30 years of age) as well as in old age (>55 years of age) people equally. VPA-induced Parkinsonism is more commonly seen in the elderly population [50]. There has been no report on the incidence of Parkinsonism in patients who use VPA for more than eight years. The actual reason as to why it does not occur in patients who use the drug for more than eight years is a topic to be researched. There has been no research done as to why VPA causes Parkinsonism.

 Based on the articles reviewed we would like to make the following hypothesis. VPA’s mechanism of decreasing seizures is by blocking neuronal activation. It does so by blocking sodium, potassium, and calcium channels across the neuronal membrane [23]. When the drug is used chronically over many months, it would cause down regulation of sodium, potassium, and calcium channels on the neuronal membrane. A decreased neuronal activation may cause a temporary physiological inactivation of the neuron. When multiple neurons are temporarily inactivated physiologically primarily in the basal ganglia of the brain, the patient develops symptoms of Parkinsonism. The above mechanism would likewise explain as to the reason behind symptoms resolving when the drug is stopped. It is because the neurons are only temporarily inactivated physiologically, which means when the drug is stopped, there is upregulation of the sodium, potassium, and calcium receptors on the neuronal and axonal membrane leading to neuronal activation and the neuronal membrane potential being the same as before.

Limitations 

We were able to find out what the risk factors for VPA-induced Parkinsonism are, the various symptoms and investigations that need to be done to differentiate between VPA-induced Parkinsonism and VPA unmasking the underlying potential for Parkinson’s disease. We were not able to identify any research in Pubmed and Google Scholar that explains the underlying mechanism as to how VPA induces Parkinsonism when taken for a long time. We were also not able to find any reports online about Parkinsonism's incidence in patients who have been taking VPA for more than eight years. There were also no reports about why this adverse effect is not dose-dependent and occurs only due to chronic intake of the drug's maintenance dose.

Future Thrust

There are many areas regarding this topic which requires further research in the future. The primary biochemical and pharmacological mechanism as to why VPA induces Parkinsonism remains unknown and requires clinical research. The fact that there is no incidence of Parkinsonism in patients who takes VPA for more than eight years remains a puzzle and needs to be researched. The fact that this particular adverse effect of VPA is not dose dependent rather the chronicity of the use also requires further research.

Valproic acid is one of the most commonly prescribed drugs by physicians. The fact that VPA may cause Parkinsonism after prolong usage should be a concern for physicians who prescribe it. VPA-induced Parkinsonism is highly under-reported. There is very little research available online regarding this topic, as evidenced by the Results section of this article.

## Conclusions

Chronic usage of VPA may cause a severe and disabling complication called Parkinsonism. A review of the literature has shown that there is an equal incidence in both males and females. It is more common in the elderly population. It is not dose-dependent as all the patients reported were taking the usual dose of the drug (800 mg/day). Patients who developed Parkinsonism had serum levels of the drug in the therapeutic range. Chronic intake of maintenance dose of VPA seems to be the cause. The symptoms, in most cases, improved over a few weeks. It fully resolved in a few months after stopping the drug. There is no benefit of dopaminergic therapy like levodopa in such patients. In cases where there is no improvement of the symptoms over many months, one must always think of VPA unmasking underlying potential for developing Parkinson's disease. Such patients benefited from levodopa therapy.

Valproic acid is one of the most commonly prescribed drugs by physicians these days. In this article, we have discussed one of the significant and most frequently overlooked adverse effects of VPA. This review is intended to aid physicians in early detection of one of the easily overlooked adverse effects of VPA. It should prompt initiation of the necessary treatment as this condition is reversible and easily treatable. This review is also intended for patients with risk factors who are on long-term treatment with VPA. It would help educate them on the various symptoms of VPA-induced Parkinsonism so that they can consult a physician at the earliest and receive prompt treatment for the same.
